# The Need to Consider Food Systems in Health-Oriented Food Policy and Programs^☆^

**DOI:** 10.1016/j.cdnut.2024.103775

**Published:** 2024-05-14

**Authors:** Alanna K Higgins

**Affiliations:** School of Geography, University of Nottingham, Nottingham, England, UK

**Keywords:** produce prescriptions, food and nutrition policy, West Virginia, food system transformation, institutional ethnography

## Abstract

Produce prescription programs (PPPs) are place-based interventions at the intersection of public health and local food advocacy. These programs have expanded significantly across the United States since 2010, particularly taking off in the state of West Virginia. This article draws on a 4-y institutional ethnography of PPP programs and associated policy. Although the possibilities of building support for improving community health alongside the livelihoods of small-scale producers is compelling, there exists an overall decontextualization from broader social and political determinants of health. This article concludes that although programs are able to meet some acute needs for program participants and provide income for small-scale producers, this decontextualization results from a lack of consideration of wider systems within policy and program construction, leading to missed opportunities for food system transformation.

## Introduction

Produce prescription programs (PPPs) are place-based interventions at the intersection of public health and local food advocacy. Advocates of PPPs argued they are designed to address a wide range of social and federal concerns around chronic disease, healthcare spending, and local agricultural economies. In addition to ostensibly subsidizing production, the focus on small-scale producers to address public health issues also arises from local food seen to be “healthier” and preventative of diet-related disease by those espousing its nutritional value [1,2]. Overall, they are seen as a way to address both food and health systems.

These proposed outcomes can be attractive to places like West Virginia (WV), where the population faces a 20% food hardship rate, low household incomes, high rates of under- or non-insurance for health, and primary care shortage areas [3,4]. They are further enticing given the state and national attention paid to the prevalence of poor health and chronic disease in WV [5].

This article draws from a wider interdisciplinary doctoral project examining the sociohistorical trajectories of discourses, practices, and social relations of federal nutrition policy and PPPs. Specifically, it examines the construction of PPPs and their implementation in WV as unit of analysis to discuss the challenges and opportunities of United States food and nutrition policy to contribute toward transforming food systems. In detailing challenges around policy formation, funding, organizational and program focus, and produce procurement, this article shows the decontextualization of program creation and implementation from the very systems within which they are embedded and that result in the problems PPPs try to address.

## Research Approach and Data Collection

This article presents findings from a 4-y institutional ethnography examining changes in federal nutrition policy alongside the organization and implementation of PPPs [6]. Data were collected from key informant interviews, participant observation, and event ethnographies to form the institutional ethnography of healthy food access programs and policymaking. Data points are regarded as an entry into the social relations that underpin these settings [7]—in this case, the construction and implementation of PPPs within WV. This approach allows us to discover and examine the assemblage and coordination of institutional knowledge affecting food and nutrition policy across different political, economic, and social relations [8,9].

Participant observation occurred between 2018 and 2022 at various sites within WV alongside event ethnographies in multiple settings [10]. There were 7 visits at 2 PPPs in northern WV, which included assistance with running the produce stall, logistical planning, and research requested by the organizations, and planning meetings. Additional data came from various planning meetings from a larger statewide group and discussions with PPP producers. Multisited event ethnographies collected longitudinal data on organizational dynamics, practices, and other events influencing the field and its knowledge practices [[10], [11], [12]]. Events included: the 2018 and 2019 Southern Obesity Summits, 2021 USDA Agricultural Outlook Forum, 2021 Consumer Federation of America Virtual National Food Policy Conference, various 2021–2022 sessions of the Virtual Food Policy Conference Series, and numerous meetings of a WV-wide multiorganization coalition^1^ focused on PPPs.

Initial key informant interviews happened between May and October 2018 with 30 program organizers across the United States^2^. Criteria for identifying key informants were: involvement in PPP organizations through either medical, administrative, or produce production work. Key informants included producers, state employees, healthcare workers, clinic administrators, State Extension agents, and community organizers. Twelve of the 30 interviews were WV specific and form the basis of this analysis together with the participant observation and event ethnographies. Semistructured interviews captured informants’ perceptions of how their PPPs operate and impact on communities. There were 49 questions regarding organizational structure, funding, program goals, participant enrollment and monitoring, organizer and producer enrollment, and perceived benefits to communities, food systems, and medical practitioners. The interviews provide insight into program goals and framing, funding structures, participant prerequisites and monitoring, producer involvement, and challenges experienced by programs and their organizers. PPP participants were not interviewed as the aim of the research was to understand organization and implementation of programming.

## PPPs in the United States

PPPs are projects focused on specific populations with a diagnosis of having, or being at risk of, certain chronic diseases considered to be diet-related. The attention to health status primarily focuses on a diagnosis of “obesity”^3^, diabetes, or hypertension. PPPs are often coordinated by numerous organizations partnering to address access to fresh produce and incidents of chronic disease. They typically include partnerships between healthcare practitioners, nonprofits promoting community development, local food, or public health, and an agricultural producer or grocery retailer. These programs are built to specifically incentivize the identified individuals to change their diets through subsidized production from local growers, or vouchers to food retail outlets. In most cases, the “prescription” includes either a voucher to purchase/acquire fresh fruits and vegetables (FVs) in grocery stores or farmers markets, or the individuals receive a weekly food box (much like community-supported agriculture [CSA]). Some programs also include nutritional and health education; in the forms of classes, participant-provider interaction, or literature. Although many current programs originate from healthcare settings, with the advent of funding from the 2014 and 2018 Farm Bills, there has been an increase in PPPs driven by community organizations or small-scale producers. PPPs are frequently presented as a “win-win-win” proposition because they address concerns about individual eating habits (particularly among low-income communities), a lack of income for small-scale farmers, and rising healthcare utilization in the United States. Oren Hesterman—founder and CEO of Fair Food Network—says that the triple win idea frames the desired outcomes of PPPs as it addresses healthcare costs, local economies, and improved diets. Many federal policy lobbyists, congressional policymakers, and program organizers currently see these programs as a way to address systemic issues around food access and negative health outcomes [6].

The rollout of PPPs has rapidly expanded across the United States over the past decade, particularly after the Affordable Care Act (ACA) in 2010 and funding allocations from the 2014 and 2018 Farm Bills. Although this federal funding exists, many PPPs are still reliant on philanthropic donations and grants (which are sometimes tied to the Community Benefit Investment provision of the ACA). A majority of PPPs have eligibility standards around income and health status that participants must meet to take part in the program. Although eligibility standards based upon medical diagnoses are the current norm, they can change from program to program, place to place, and doctor to doctor.

PPPs across the United States have a mix of programmatic goals, including concerns over chronic disease rates and rising costs of healthcare, the promotion of community health and wellbeing, improving relationships between healthcare establishments and communities, addressing food insecurity, and the desire to advance local food systems. In fact, proponents often claim that local food production is not only better for the environment, but also better for a person’s health. A small percentage of programs across the United States are less focused on diagnosis and instead center around food insecurity, such as the one program run out of a food bank in Pennsylvania. Another program in Ohio used a 2-question food insecurity screening tool for participant eligibility [14].

However, these programs are few and far between, as most PPPs focus on chronic disease [15]. As such, the outcomes that programs focus on revolve around participant engagement and biomedical screening. Weight and BMI are frequently used, along with blood tests to measure Hemoglobin A1C or a lipid panel. These tests can be administered before, during, and after the program. Medical practitioners and program organizers have often equated lowering A1C levels in participants as a key indicator of program success and by extension, proof that PPPs are a way to lower healthcare costs.

Although medical diagnosis is the most common variable, PPPs across the United States have a mix of programmatic goals, including concerns over chronic disease rates and rising costs of healthcare, the promotion of community health and wellbeing, improving relationships between healthcare establishments and communities, addressing food insecurity, and the desire to advance local food systems. The current landscape of cross-cutting goals, funding streams (and their associated obligations), and operationalization reveal tensions within the institutional relationships of PPPs and the current missed opportunities for food system transformation.

## West Virginia FARMacies

Within WV, PPPs are primarily known as “FARMacy” programs. The first FARMacy started during 2016 in the city of Wheeling. Throughout the state, the reception and atmosphere surrounding these programs has been enthusiastic and positive. In fact—echoing similar phrasing from national-level discussions—PPPs are called a “win-win-win” by almost everyone involved from the state Commissioner of Agriculture to healthcare practitioners, politicians, and small-scale producers. The programs have reached a level of popularity that the WV Department of Agriculture regularly receives calls asking if a program will be started in the person’s area.

Since 2016, PPPs have quickly expanded across the state and now operate in 18 counties. These programs are coordinated by groups of healthcare partners, growers and produce aggregators, and community and state organizations. As programs have developed across the state, their focus has stayed relatively the same—targeting low-income, food-insecure communities, with a strong focus on chronic disease, diet, and biometric collection. To date, program expansion has been greatly facilitated by large grant funding, which has allowed for the extension of FARMacy into new areas.

Organizational coordination varies within FARMacies, and was dependent on program planning, logistics, and staffing (through both paid and volunteer labor). The number of organizations involved in a program varied anywhere from 2 to 8 entities responsible for coordination and implementation. This included a range of different organizations working together (such as medical practitioners or clinics, community groups, and local producers), or a single organization driving and implementing the program. Interestingly, when those involved in a FARMacy were asked about the organizations partnering to carry out their specific program, the names and numbers of organizations differed. Organizations that were sometimes left out included those who provided services such as connecting programs to producers, or those running the nutrition education components. Recently, a majority of FARMacies within WV have operated under a coalition of organizers from public health professionals, medical practitioners, small-scale producers and produce aggregators, and health and nutrition educators.

The goals for all WV FARMacies broadly follow the medical model of PPPs, focused on individuals with a diagnosis of chronic diet-related disease. This has led to specific metrics for participant eligibility, with programs basing these requirements on a certain income bracket, lack of health insurance, and having certain health diagnoses. Sometimes eligible participants were “baked in” to the program, particularly if the FARMacy is run out of a Federally Qualified Health Center, which already administers to those who are under- and uninsured. A majority of programs also include activities such as educational classes or literature, recipe and cooking demonstrations, and sometimes produce tastings.

## Transformation and Justice Within Food Systems

Academic, policy, and community interest in food systems has expanded since the early 2000s—however, there are many ways the “food system” is conceptualized, from a focus on types of agricultural production to socio-ecological systems, sustainability metrics, and “industrial” compared with “traditional” [16]. Despite inconsistencies in conceptualizations and definitions, the belief that food systems need to change is ubiquitous. The goal of food system transformation is to “empower” people and communities toward food systems that nurture both land and people [17].

This article follows the view that food systems are intricate arrangements of “interdependent structures” which not only cover activities like food harvesting, eating, and waste, but additionally contend with power dynamics and resulting social and political structures [18,19]. It also mirrors calls for food system transformation to be holistic, with “a broad definition of sustainability that covers not only environmental sustainability, but also the social dimensions of equity, security, sovereignty, access, justice, and the right to food and fair livelihoods for all” [20]. However, without the input of all those involved in food systems—and indeed, those currently marginalized and oppressed within broader political and social systems—any food or agricultural policy will be disjointed and antithetical to a *just* food systems transformation [21].

Just food system transformations have the potential to address economic, environmental, and health challenges—but must be vigilant to not romanticize solutions or elide larger structural reasons environmental degradation, food insecurity, and negative health outcomes exist. When thinking about food systems, policy, and health, we must remember to focus on the *politics* of food rather than just advancing policy [22]. Caraher and Coveney [23] argue the current approach to food policy ignores the food *system* with its focus on individual, biomedical understandings of diet and health. This includes the growing set of discourses and interventions over the past 20 y centered on addressing health inequities through increasing access to local food [24]. Although this intersection of local food and public health can seem promising, Muller et al. [25] note that “the complexity of the food system creates challenges for identifying and incorporating health-supporting policy opportunities.” For example, despite the claims that local food initiatives are a panacea to negative environmental, social, and economic change, there is little empirical evidence to support those assertions. In fact, much of the discourse around food system transformation toward the “local” disregards that local production is also subject to power imbalances and dynamics also found in industrialized agriculture [26,27]. Furthermore, local production is not inherently devoid of pesticide or chemical use and can occur in places with high environmental contamination. This includes WV, with its history of water and land pollutions from coal mining, mountaintop removal, and chemical spills [[28], [29], [30]].

This decontextualization from wider structures and a singular focus on individuals rather than systems creates a disconnect between public health interventions and policies and the actually existing food environments [31]. Jetter and Cassady [32] point out that interventions and policies—such as nutrition education programs—remain ineffectual because of their disregard of the food environment of the areas they work in. Caraher and Cowburn [33] discuss how debates around the burden of diet-related diseases tend to operate on a binary; one end arguing for change in individualistic behaviors like shopping and eating, with the other end contending that diseases also result from structural causes like environmental or racial injustices. Lyson [34] concurs, observing that the paternalistic efforts of policies around food and health need to be aware of class and race issues while also reframing debate away from the current binary.

## Decontextualization within United States PPPs

The creation and implementation of PPPs and associated policy currently reproduce this decontextualization of health-oriented food policy away from thinking about food systems and toward individualistic interventions. There are several issues which contribute to this, some of the largest being how policies are formed and what they appropriate money toward, organizational tensions within PPP programming, and a lack of integration of producers in the planning process coupled with differences in produce provisioning.

## Policy Formation and Funding Opportunities

Current policies that fund PPPs focus on the medical model of the programs. The 2018 Farm Bill included the Gus Schumacher Nutrition Incentive Program (GusNIP), which “bring[s] together partners from various parts of the food and healthcare system to help improve the health and nutrition status of participating households” [35]. Specifically, within GusNIP is a set of funding for PPPs to develop and evaluate these projects. Like most others, GusNIP-funded PPPs have eligibility metrics for what individuals can partake in the project. They are eligible to participate if they meet the following 3 criteria: they are eligible for the Supplemental Nutrition Assistance Program (SNAP), they are eligible for Medicaid, and they are “a member of a low-income household that suffers from, or is at risk of developing, a diet-related health condition” [36]. Although biometric data collection is not a specification of this funding, 1 key informant mentioned that the way the request for applications is written and the evaluation that the National Institute of Food and Agriculture focuses on promotes the collection of this data. This focus on biometrics and individual dietary patterns within current policy follows the decontextualization from wider systemic and political and economic structures at work in society within similar food and health interventions [[37], [38], [39], [40], [41]].

Although WV FARMacies directly studied for data collection were not GusNIP-funded at the time, many programs within the state have secured funding under GusNIP for nutrition incentive projects. Additionally, although the specific federal funding of PPPs did not start until 2018, the construction of this policy (and its focus on individual behavior) followed the work of past PPPs operating in other areas of the country [6]. Their work also influences the wider funding landscape that WV FARMacies rely on.

Funding and labor were a continual source of apprehension and strain within program organizing and implementation. Most funding for PPPs in WV come from philanthropic dollars, with the terrain of programming shifting each year because of the funding sources and organizational capacity. As with many other privately funded PPPs across the United States, WV programs have relied on a patchwork of funding from different sources. This has included money from large philanthropic donors like the Claude Worthington Benedum Foundation or Walmart Foundation, and grants from federal and state agencies such as the Health Resources and Services Administration. Additionally, some PPPs operated out of Federally Qualified Health Centers, which used a portion of their operating budgets toward the program.

This funding primarily paid for the produce, either through direct payment to the producers or funding the vouchers that the producers were reimbursed through. The amount of funding played a large role in the length of program and amount of produce each participant received. FARMacies in WV run an average of 15–20 wk, throughout the North American summer growing season of June through September or October. Some programs were able to provide FVs throughout this period, whereas others gave a one-time voucher. Issues of sourcing, FV production, and related funding concerns continues to be an issue for FARMacies because of the existing capacities of specialty crop producers. An example of this came toward the end of the summer 2018 growing seasons when several organizations came together to discuss starting a new program in WV’s northern panhandle. Despite enthusiasm from healthcare providers, producers involved in other FARMacy programs, and community organizers, the program never got off the ground because of a lack of production capability coupled with concerns about funding. As one organizer put it: “it’s never a problem on the other side – the problem is getting the food.” Furthermore, programs in other locations across the state had issues continuing from 2020 into 2021 because they could not aggregate enough FVs to support a program. Another aggregator, although having relative success in other fronts, was not able to get enough producers on board to create a production stream for FARMacy, and a separate program had funding but was not able to operate because there were not enough growers able to produce for the program.

A handful of programs were able to use funding for program administration and implementation, but this was not the norm. Therefore, many PPPs relied on volunteer labor from nutrition educators, community organizers, and clinicians along with in-kind donations such as physical space (for storage or program activities), or material goods or services (such as mileage, transportation, tables, tents, etc.). In fact, the lack of funding for administrative labor became a source of tension with some partners spending more time than contractually obligated. For example, 1 set of FARMacies list a total $20,820 of in-kind support (from both clinic staff and other organizations) as a part of their program costings. One nutrition educator explained that they were spending more time than allotted—at the expense of their other work—“in order to keep the program afloat and working.” One program was able to secure funding for a FARMacy manager who oversaw logistics, reimbursements, pickup days, and other arrangements. Although this program only lasted 1 y because of funding challenges, the manager position was often cited as a reason for its success, whereas those who did not have this role in their program cited the challenges behind not having a coordinator to take care of those duties, and having that central person is a “key factor” to a programs’ longevity.

Another tension around funding included which organizations applied for grants, and whether they did so individually or with other groups involved in FARMacies across the state. Although this issue is discussed here, specific names and events have been left out for confidentiality. The first example occurred in a PPP that was primarily run through 2 organizations. One organization found out that another had applied for, and received, funding without telling the other organization. This has happened more recently, with a multiorganization group working toward rolling out PPPs statewide receiving news that another organization who they have not previously worked with applied for and received funding to implement several programs. Although these 2 groups have come together to discuss implementation, this caused friction as the first group sees themselves as the progenitor of PPPs in WV. Interestingly, these strains did not exist with the state-level organization in charge of nutrition education. Because they have their own money outside of FARMacy, program organizers did not quarrel with them as there was no conflict over funding. Additionally, because of their relationships within FARMacy organizing but outside of funding relations, this group was often asked to step in to convene discussions, as they did not have any financial stakes in the proceedings.

Funding cycles were often cited as a programmatic barrier. The timing of disbursement did not always match program calendars, with money coming after the start of a program or when producers need to start planning their production, or notification of grant awards coming after a time period where participants should be recruited. In addition to these issues causing problems in setting up programs, it also resulted in complications of spending down money by the end of the grant. However, the funding for produce made a positive impact on producer incomes to some degree. Although many producers in WV are motivated through a desire to grow healthy food for their families and communities [42], most producers participating in healthy food access programs have discussed the financial instability of farming, which has dually hindered their ability to participant in programs and sometimes negatively impacted their livelihoods.

Although contemporary policy and PPPs are discussed as ways to address health issues, local food systems, and food insecurity, their current construction and operationalization does not take a food *systems* approach. As discussed in the following sections, difficulties around organizational tensions, lack of producer integration into PPP planning, and different ways of sourcing produce lead to missed opportunities for food system transformation.

## Organizational and Program Focus: Food Insecurity, or Poor Choices?

Another issue is the occurrence of tensions within the organizing and implementation of PPPs. The largest organization tension within WV PPP construction and implementation was different focus from organizers—those who solely focused on chronic disease, whereas others wanted the programs to acknowledge wider systemic issues around food and health. A large grant funded program was premised on working with an academic office to determine if healthcare providers were screening for social determinants of health, specifically using the Hunger Vital Sign [14]. However, after 2 y of grant execution, there were numerous people—both within and adjacent to FARMacies—that did not think questions of food insecurity needed to be a part of the program. In particular, healthcare providers did not see it as a necessity to ask the Hunger Vital Sign within screening participants for FARMacy. One individual involved in FARMacy organizing shared with me that most people who ran the programs did not use the food insecurity screener or other similar tools and “enrolled whoever they wanted”—though importantly who they wanted were people with specific diagnoses or specific levels of biomedical tests like Hemoglobin A1C. This person also disclosed that it was not just an issue between individual healthcare providers, but that larger organizational relationships between clinics, funders, and researchers hinged on how the programs were framed and implemented.

The lack of engagement with structural issues around food access and health were also brought up by a grower heavily involved in PPP organization, who discussed a medical professional who claimed that their patients could access free food but “chose” not to, so why would they give their patients more food. The grower referenced that these professionals did not understand “what is really a challenge out there.” Nevertheless, although program literature on FARMacy (including pamphlets, policy memos, and presentations to various outlets) continually cite issues of food access and insecurity within WV, program coordinators continue to emphasize medical diagnoses and tracking health data over issues of poverty.

However, although organizers lamented the lack of structural response to issues of poverty, health, and hunger, they had their own preoccupations with the eating and purchasing habits of people across WV, and PPP participants more specifically. An example of this occurred at one of the program’s kick-off day, where a health practitioner tied to the program told me that “my patients absolutely buy crap with their food stamps.” This statement was surprising, as this myth around SNAP use on foods considered to be unhealthy has been investigated by the Food and Nutrition Service, and shown to not be the case [43]. Many program organizers discussed societal issues in WV around lack of income and access to fresh food—but this was almost a caveat in discussions around eating habits and willingness. Healthcare providers and growers continually made comments throughout pickup days or planning meetings about how it was “tough” in WV because nobody wants to eat FVs, or that the high prevalence of certain disease states was “in large part because of what they eat.” After the 2018 FARMacy season ended, 1 grower discussed with me that FVs were attainable and it was easy for people to say they do not have money, but it was down to the fact of people going for “easy food” and not eating fresh FVs. They went on to discuss eating the right kind of food was key for changing health metrics and losing weight “but that’s a conversation [people] don’t want to entertain.” These discussions around willingness were further echoed in remarks around people’s motivations for participating in FARMacy, and that unless the participants came “to the table with the commitment [to improve health], it makes it difficult and it’s just them getting free food and that’s not a FARMacy, that’s something different.”

Since the completion of the doctoral project [6] there have been efforts from a handful of FARMacy organizers to introduce a contextual and critical reflection into program operations. Within meetings, individuals have raised questions around dignity, choice, and wider issues of poverty when discussing why programs need to expand across the state and how they should be run. Repeated appeals for the importance of food security screening occurred within meeting discussions and e-mail threads. Additionally, there have been several presentations and discussions around the social and *political* determinants of health, and their applicability to FARMacy programming. One such attempt was the creation of a Continuing Medical Education (CME) course. The CME ran through a state university, and was designed to address the “disconnect between providers and people who are operating programs”, particularly the fact that “some partners are not using the food access question/metric because there’s no resources they have or know of which can address that” issue.

## Producer Integration and Produce Provisioning

An essential part of any PPP is the provisioning of FVs. Although PPPs do not inherently need to include local produce, the programs within WV strove to do this as much as possible. Produce sourcing and distribution were dependent on funding amounts, but also the availability of specialty crop producers and produce in areas of FARMacy operations. Additionally, distribution models and produce sourcing strategies differed with programs that had higher budgets using aggregators and those with lower budgets sending participants to local farmers’ markets. Most FARMacies have a CSA-style model, where participants get a box of produce from a central pickup point. This CSA-style was a conscious choice of most program organizers—to elicit a “farmer’s market feel” for the participants, as well as maximize the amount of time growers and healthcare providers had with participants.

Production strategies differed among FARMacy producers, which also influenced differences in produce provisioning among programs. Producers in 1 program primarily used high tunnels in their operations, which allowed them to grow certain varieties of produce earlier than with normal field planting methods. Other producers in the region were sporadically turned to in the event of production problems or to bring in products the core group did not grow. The rationale behind this was to use different vendors from the local farmer’s market that could not supply FARMacy on a regular basis and develop a niche market for them. It is important to note that high tunnels are not prevalent for all growers in WV and require not only the land and capital for construction, but also additional knowledge of navigating bureaucracy around building permits. Therefore, although the growers in this program were able to cooperate and utilize FARMacy as another market to sustain their livelihoods, this was also predicated upon their existing on-farm capital and access to other resources. Although beneficial for the producers who can access these resources, those who cannot—which encompasses the majority of small-scale producers in WV [42]—are unable to benefit from engagement with PPPs in their areas.

Other FARMacy programs in WV had growers who either did not have access to these resources or a cooperative growing relationship. For instance, the aggregator for another program brought in produce every week which they had either grown themselves or sourced from other areas at the time. This included sourcing produce from an auction across state lines—a fact that was referred to as a “dirty little secret” from 1 organizer and was not widely advertised, but was openly discussed by the aggregator in multiple conversations and planning meetings. Although the producers in the first program had success with their grow plan, the aggregator for the other mentioned that it was a challenge to make sure they were offering enough of a variety of produce and “it can be hard to acquire or grow a high enough volume of a lot of other things” besides the cucumbers, zucchinis, and tomatoes which are common at that part of the growing season.

Sourcing produce and fruit and vegetable production continues to be an issue for FARMacies because of the existing capacities of specialty crop producers. For instance, several organizations came together at the end of summer 2018 to discussion starting a new program in WV’s northern panhandle but because of a lack of production capability (and funding concerns) the program never got off the ground. As 1 organizer put it: “it’s never a problem on the other side – the problem is getting the food.” Furthermore, programs in other locations across the state had issues continuing from 2020 into 2021 because they could not aggregate enough FVs to support a program. Another aggregator, although having relative success in other fronts, was not able to get enough producers on board to create a production stream for FARMacy, and a separate program had funding but was not able to operate because they could not get enough growers able to produce for the program. To head off such challenges, 5 main producers and a hired manager made up the core of program organizers, with their coordination efforts creating a proto-farmers’ cooperative in 122 the process. Before the growing season, the producers sat down to draw up grow plans, designating who was growing what items, when the items were expected to be harvested, and price points for reimbursement. Within the grow plan, high value crops were distributed as equally as possible, so that both growing responsibilities and profits were distributed across the group. The growers’ organization the producers belong to kept 10% of the market price, with producers bringing home the remaining 90%. One producer told me that it was “amazing” to have a 5%–6% increase in income, along with being very happy about the scheduled grow plan and constant communication among the main producers. Another producer noted that FARMacy gave them an immediate outlet and they did not have to worry about holding onto unsold product. Producers also experienced other benefits from their participation, such as increased exposure to the public, assistance in writing grants, and leveraging their FARMacy experience for other funding. As an organizer at a third site stated: “I think this speaks to how this impacts local growers because it can help them expand and serve additional people in the future.”

However, this integration of producers into FARMacies, or any other PPPs, is not the norm. Although federal policy around PPPs and program organizers often tout the importance of these programs for the local food economy, at present the majority of programs are coordinated by healthcare or community groups with producers brought in ad hoc. This leads to issues of procuring produce, as many producers plan their seasons well in advance—not only what they are growing, but where they plan on selling or distributing the produce. For PPPs to successfully provide enough produce (and a variety of it), there needs to be producers sitting around the table while programs are thought out.

## Discussion

There are a number of challenges and complexities in ways that PPPs are being organized and implemented. This leads us to considerations around the conceptualizations of what problem(s) FARMacies address. This is another area of challenges and complexity, with the discourse of healthism conflicting with that of food access. Viewing PPPs through a healthism lens [44,45] influences the way in which bodily health is measured, decontextualizing the broader contexts which influence individual and community health. This decontextualization removes discussions of broader influences on health—such as racism, classism, and sexism—from deliberations about disease and illness. This decontextualization is important to consider not only in overall program organization, but also in relation to the health of West Virginians. Although PPPs ostensibly put fresh produce into the hands of some who need it, they can reify stereotypes and harmful assumptions from policy makers, community organizers, and health practitioners [15]. These include a narrow focus on behavior, instead of reckoning with systemic issues like poverty and unemployment, environmental pollution, and the racialization of bodies, which have clear effects on both individual and population health. Relying on a medical diagnosis combined with the paternalism some healthcare practitioners and growers displayed around “willingness” to eat produce constructs these programs within beliefs of the deserving poor compared with the underserving poor—those who society feels should be aided by the government because they are in certain circumstances and those they feel are “lazy” [46,47]. This belief was exhibited by program organizers who lamented that participants had health issues and could not access fresh food but in almost the same breath would talk about how participants were getting “free food”. This leads us to question why it is healthism—rather than food access—which is seen to compel organizations to create and carry out FARMacy. The lens of healthism helps us to interrogate these questions and potentially explain why PPPs are becoming increasingly popular across the United States, particularly here in the Mountain State.

Apprehensions around funding is another complexity, which could turn into a significant challenge if not rectified. Regarding the current funding situations, the reliance on patchwork funding—although currently necessary for program implementation—can cause convoluted flows of resources, wherein 1 organization receives a grant but then sends all or a portion of it to another organization for program administration or produce. The existence of patchwork funding is not caused by the FARMacy organizers, but has been highlighted as a main reason for program precarity. Without consistent and substantial funding, these programs do not always exist from 1 y to the next, changing their geographic distribution. Additionally, funding issues also caused constraints around how long programs are able to operate. Although 15–20 wk may seem like a long time, it is actually very short when considering issues of hunger, food access, and negative health outcomes. Furthermore, 15–20 wk is not substantial enough to make long-term changes to an individual’s health status, nor necessarily have the longitudinal data to ascertain changes in health outcomes or if outcomes are because of FARMacy and not other factors.

Apropos of the intended effect of programming on individual and population health, these issues constrain the ability of FARMacy to make long-term effects on health outcomes and food access. The constraints around what funding was for also caused issues around the distribution of labor among organizations and particular individuals. The relative success of Program #1 shows that having a central FARMacy administrator assist in program operation goes a long way in making the program a success. Additionally, although grant procurement is important to the longevity of certain organizations, the tensions around application for and competition over grants affect working relationships between individuals and organizations. Nevertheless, questions arise around these funding tensions and the greater good for program participants who will be able to access FVs. And though funding is a serious issue within the existence and implementation of programs, the distribution of money also has clear impacts on the livelihoods for WV producers. If long-term, stable funding is able to be secured and distributed in a way that producers can plan for increased production and potentially use funding to access capital and infrastructure, then FARMacy does have the potential to influence local agricultural economies.

Although this article has outlined several areas of friction within FARMacy programming, I would like to end with a short discussion of the possibilities FARMacy brings. Within the state, access to food and healthcare is a serious predicament for a majority of the population [48]. Although these compounding problems are rooted in systemic obstacles, PPPs have the opportunity to address acute needs through the distribution of produce and increased contact with healthcare professionals. Many of the participants in WV FARMacies experience barriers in seeking healthcare—whether through issues of topography, time, or more likely because of low incomes and being under- or uninsured. Additionally, in a state where many do not have the income or physical access to fresh foods a weekly box of produce or voucher for FVs does wonders for providing nourishment and security for individuals and families. Therefore, the increased time with a healthcare provider and the increased (albeit time restricted) access to FVs arguably address some of these needs. However, to help FARMacy programs—and PPPs more broadly—there are several recommendations for program construction and implementation. The first echoes the arguments that Barnidge et al. [49] have already made around incorporating food as a right into the “food as medicine” movement. To address the systemic reasons behind negative health outcomes and lack of produce access, we need to think about “a politics of the possible” when it comes to food and nutrition policies and programs [50,51].

Additionally, initiatives themselves can make programmatic changes to consider and potentially address wider issues of poverty, health, and food access. This includes recognizing that it is not individual behavior which creates negative health outcomes, but that these behaviors are a result of the wider systems and environments that people live within. For produce prescriptions—especially WV FARMacies—this is incredibly important, given the histories between extractive industries and labor practices on wealth distribution and thus access to healthcare and food. Additionally, programs would benefit from incorporating an environmental perspective into their priorities. Not only does environmental pollution and soil quality affect human health, but it also affects the quality of food being grown. This is another important aspect WV programs need to consider again given its history of water and land pollution from coal mining, mountaintop removal, and chemical spills. Programs can also include an evaluative approach which may assess whether program arrangements and outcomes are reinforcing systems that initiatives may be trying to change, and who is doing the problem definition and program construction. Furthermore, this evaluative approach links to questions of reproduction of harms within program focus—particularly the trap of the weight-centered paradigm [52]. For this, we turn to Marquisele Mercedes who argues that “The existence of differential access to food is the problem and is evidence of a longer history of inequality that should be the primary concern of all working in the food equity space” [53].

In conclusion, the current implementation of PPPs within WV is a complex landscape of organizational connections, points of tension, and areas of possibility. Currently, PPPs and the policies appropriating federal money to them currently lack consideration of the *system* within the food system. Although public health advocates have sought to engender the “systems change” conversation through the construction and implementation of these programs, advocates in WV who come together to coordinate PPPs also struggle with the binary of individual health intervention or systemic and structural change. PPP organizers actively participate in many of the signifying practices critiqued by the literature and although they are indeed focused on behavior change in individuals, broader changemaking is on their minds. However, in practice they confront significant contradictions that limit their efforts to go past construction of interventions “and into the systems that create the structures in which we work, live and play” [54]. The resulting tensions that emerge in practice between producers, public health advocates, and health practitioners exist within the overall discordance between what current federal policy is trying to achieve and what the outcomes are. This disjuncture shows that systems work is not so simple, as it is constantly negotiated and carried out within the very frameworks it is trying to improve or rectify. However, the delineations of program focus attributed to these discourses still deciphers the “problem” and then decides who receives—who deserves—resources.

Nevertheless, there are also clear benefits to their implementation. Given appropriate support and funding, FARMacy programs have benefits for the businesses of local specialty crop producers and their livelihoods. And there is no question that these programs—in WV and further afield—are attempting to address issues within food and health. However only providing a certain number of people produce for what is actually a short period of time does not address the systematic reasons behind why these inequities exist in the first place, nor does it “move the needle” on the prevalence of certain negative health outcomes. Questions arise when we dig into the use of health status as eligibility and biomedical metrics as indicators of “health” and “wellbeing.” As 1 nutrition educator put it when discussing the lack of attention to food insecurity: “Why aren’t [they] trying to enroll the people who *need* the food?”

## Author contributions

The sole author was responsible for all aspects of this manuscript.

## Conflict of interest

The authors report no conflicts of interest.

## Funding

Funding for this dissertation was provided by a West Virginia University Provost Fellowship, WVU Department of Geology and Geography and Eberly College Travel Grants, a Graduate Association for Food Studies Travel Grant, a WVU Geography Program Graduate Enrichment Award, a Take Back Our Health WV! Fellowship, a West Virginia Science and Technology Policy Fellowship, and funding from the WVU Food Justice Lab and Center for Resilient Communities.

## Data availability

Data described in the manuscript will be made available on request.

## Footnotes

1 Organizations’ names have been kept confidential, but include university Extension, healthcare systems and nonprofits, academic units and individuals, regional growers’ groups, and food system non-profits.

2 Key informants came from the following states: West Virginia (12), Virginia (6), Tennessee (3), Pennsylvania (2), California (2), Vermont (1), Delaware (1), Oregon (1), Ohio (1), and Texas (1).

3 The word “obesity” is placed in quotations (except for direct quotes) to signify its pathologizing of, and creation of fear around, fat bodies [13].
